# Intestinal Sirtuin 1 Controls Liver Regeneration via Regulating the Intestinal Farnesoid X Receptor/Fibroblast Growth Factor 15 Axis in Mice

**DOI:** 10.1016/j.jcmgh.2026.101813

**Published:** 2026-05-22

**Authors:** Sian Seaman, Paula Ruiz, Vinícius Dias Nirello, Laura Martin-Castilla, Pablo Elizalde-Garcia, Adriana Vega-Zuza, Lucia Vegas-Petrina, Cristina Lumbreras-Perales, Mark Philo, Mar Moreno-Gonzalez, Patrick Varga-Weisz, Naiara Beraza

**Affiliations:** 1 Food, Microbiome and Health Institute Strategic Programme, Quadram Institute Bioscience, Norwich Research Park, Norwich, United Kingdom; 2 Department of Genetics, Evolution, Microbiology, and Immunology, Institute of Biology, University of Campinas, Campinas, São Paulo, Brazil; 3 School of Life Sciences, University of Essex, Colchester, United Kingdom

**Keywords:** FGF15, FXR, Gut–Liver Axis, Liver Regeneration, Sirtuin 1

## Abstract

**Background & Aims:**

The contribution of the gut–liver axis during liver regeneration remains poorly understood. Understanding the mechanisms underlying this process is critical for developing novel therapeutic strategies for liver diseases and transplantation. Here, we aimed at defining the role of intestinal sirtuin-1 during liver regeneration in mice.

**Methods:**

We performed partial hepatectomy to intestinal-specific sirtuin-1 knockout mice (SIRT^intKO^) and wild-type littermates. Immunostaining and immunoblotting were performed on liver and intestinal tissues to assess proliferation and senescence, as well as farnesoid X receptor and fibroblast growth factor-15 protein expression. Bulk RNA sequencing was performed on liver tissues.

**Results:**

We found that SIRT^intKO^ mice had significantly reduced hepatocyte proliferation and increased hepatocyte senescence, accompanied by accumulation of bile acids in the liver that was associated with profuse parenchymal damage. Still, SIRT^intKO^ restored liver mass at comparable levels to wild-type mice at 10 days after partial hepatectomy, which was accompanied by the activation of liver progenitor cells in the livers of knockout mice. Transcriptomic analysis of bulk RNA sequencing data from liver tissue samples at the priming (6 hours) and proliferative phase (24 hours) after partial hepatectomy highlighted that impaired hepatocyte proliferation in SIRT^intKO^ mice coincided with the downregulation of the signal transducer and activator of transcription pathway and disruption of amino acid and lipid metabolism. Mechanistically, intestinal sirtuin-1 depletion was associated with reduced expression of farnesoid X receptor and fibroblast growth factor-15 in the small intestine. The intestinal-specific activation of farnesoid X receptor with fexaramine treatment successfully re-established hepatocyte proliferation and restored the liver parenchyma integrity in SIRT^intKO^ mice.

**Conclusions:**

Intestinal sirtuin-1 is a key regulator of liver regeneration through upstream control of the farnesoid X receptor/fibroblast growth factor-15 axis following partial hepatectomy.

## Graphical abstract

**Figure:**
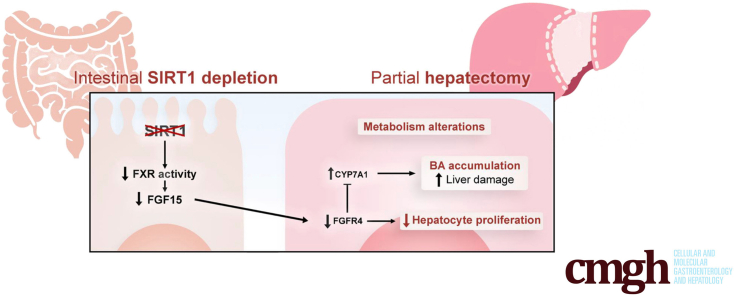

**Summary**

Intestinal sirtuin 1 regulates liver regeneration by controlling the farnesoid X receptor/fibroblast growth factor 15 axis. Intestinal sirtuin 1 deletion disrupts bile acid homeostasis, impairs hepatocyte proliferation, and increases liver injury and senescence. Intestinal farnesoid X receptor activation restores regenerative capacity and liver integrity in mice after partial hepatectomy, supporting that sirtuin 1 is an upstream regulator of the farnesoid X receptor/fibroblast growth factor 15 axis.

**What You Need to Know**

**Background**

The contribution of the gut-liver axis during liver regeneration remains poorly understood. Sirtuin 1 is a key metabolic regulator, yet its role in controlling farnesoid X receptor/fibroblast growth factor 15 signaling during regeneration is unknown.

**Impact**

Intestinal sirtuin 1 controls liver regeneration via the farnesoid X receptor/fibroblast growth factor 15 axis. Loss of intestinal-specific sirtuin 1 promotes bile acid-mediated liver injury and impairs hepatocyte proliferation, identifying a novel, targetable gut-liver signaling mechanism.

**Future Directions**

Future studies should validate these findings in humans and explore the therapeutic modulation of intestinal sirtuin–farnesoid X receptor signaling to enhance liver regeneration in clinical settings such as resection and transplantation.

Liver regeneration is a coordinated cellular response that allows the liver to restore its mass and function after resection (partial hepatectomy [PHx]). The ability of the liver to regenerate following PHx is well-described, with the activation of hepatocyte proliferation as the main cellular event to restoring the liver mass.^1^

Bile acids (BAs) are crucial mediators of liver regeneration. Previous studies have demonstrated that BAs act as signaling molecules that regulate hepatocyte proliferation during liver regeneration after PHx.^2^

Farnesoid X receptor (FXR) is the master regulator of BA metabolism. BAs activate FXR, which, in turn, suppresses their synthesis, thus preventing hepatic toxicity. The regulation of BA synthesis is a key example of the gut–liver axis communication. BAs synthesized in the liver are secreted into the small intestine postprandially where they activate the FXR–fibroblast growth factor 15 (FGF15) pathway that inhibits BA synthesis in the liver by inhibiting cholesterol 7 alpha-hydroxylase (CYP7A1) via FGF15–fibroblast growth factor receptor 4 (FGFR4) signaling.^3^^,^^4^

FXR has been shown to contribute to liver regeneration after PHx. Huang and colleagues demonstrated that systemic deletion of FXR in constitutive knockout (KO) mice led to decreased hepatocyte proliferation after PHx due to dysregulated BA homeostasis, although FXR^−/−^ mice reached similar liver mass at later stages after resection.^2^ Further work by Zhang and colleagues dissected the liver- and intestinal-specific role of FXR in controlling liver regeneration.^5^ Hepatic deletion of FXR led to attenuation of hepatocyte proliferation and delayed liver regeneration,^5^^,^^6^ whereas intestinal FXR deletion also contributed to defective hepatocyte proliferation via the activation of FGF15 expression after PHx.^5^

The essential role of FGF15 in controlling liver regeneration was further established by Uriarte and colleagues, showing that FGF15 KO mice had increased mortality and liver damage due to increased liver BA levels and reduced hepatocyte proliferation after PHx.^7^ Further work supported the relevance of FGF15–FGFR4 signaling in mediating liver regeneration in mice.^8^

The role of sirtuin 1 (SIRT1), a nicotinamide adenine dinucleotide–dependent class III histone deacetylase, in regulating FXR activation has been previously described.^9^, ^10^, ^11^ SIRT1 promotes FXR activation by deacetylating FXR, counteracting p300-mediated acetylation.^9^ Reduced SIRT1 activity leads to persistent acetylation of FXR, which impairs DNA binding and reduces transcriptional activity. We and others have demonstrated that both the overexpression of SIRT1 and the hepatocyte-specific deletion of SIRT1 impair FXR activation and function, leading to increased susceptibility to liver injury.^10^^,^^11^ Previous work in intestinal-specific SIRT1-deficient mice has supported that SIRT1 also regulates FXR in the intestine, controlling systemic BA metabolism.^12^

In the context of liver regeneration, both SIRT1 hepatocyte-specific deletion^13^ and SIRT1 overexpression have been shown to reduce hepatocyte proliferation after PHx,^14^ supporting the role of SIRT1 in regulating liver regeneration.

However, the detailed role of intestinal SIRT1 in regulating liver regeneration has not been defined. A better understanding of how intestinal signaling pathways control liver regeneration may provide relevant cues to develop potential strategies for enhancing liver regeneration in disease and postsurgical contexts.

## Results

### Intestinal Sirtuin 1 Deletion Leads to Increased Liver Tissue Damage and Reduced Hepatocyte Proliferation After Partial Hepatectomy

**Figure 1:**
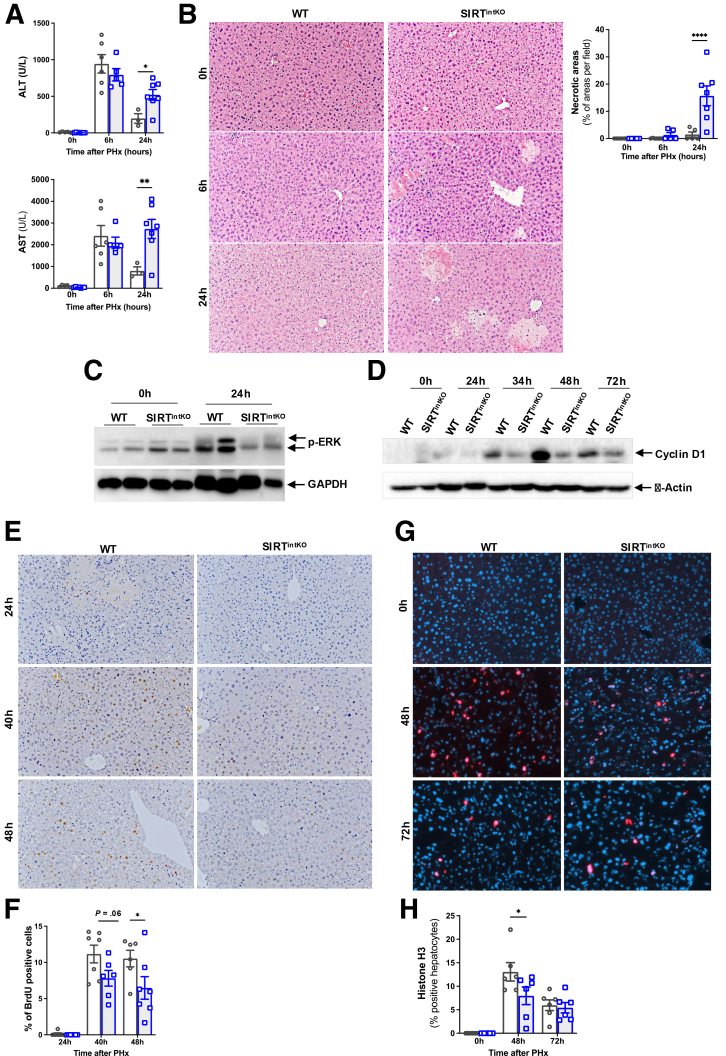
**Intestinal-specific SIRT1 deletion leads to increased liver injury and attenuated hepatocyte proliferation and mitosis after PHx.** (*A*) Serum transaminases ALT and AST in serum samples and (*B*) hematoxylin and eosin staining and further quantification of necrotic areas on liver tissue slides showing increased liver damage in SIRT^intKO^ mice compared with WT littermates at 0, 6, and 24 hours after PHx. (*C*) Western blot analysis of liver tissue samples showing differential phosphorylated ERK1/2 and (*D*) cyclinD1 expression in WT vs SIRT^intKO^ mice at different timepoints after PHx. (*E*) IHC showing BrdU-positive hepatocytes and (*F*) quantification of the percentage of positive cells at 24, 40, and 48 hours after PHx in WT vs SIRT^intKO^ mice. (*G*) Immunofluorescence showing p-HistoneH3–positive cells and (*H*) quantification of the percentage of positive cells at 0, 48, and 72 hours after PHx in WT vs SIRT^intKO^mice. Analyses were done from n = 3–7 mice. Representative images are shown from 10× (*B*) and 20× (*E* and *G*) magnification. Values are mean ± standard error of the mean. Statistical differences were determined using 2-way analysis of variance with Holm–Sidak correction. ∗*P* < .05, ∗∗*P* < .01, ∗∗∗∗*P* < .0001.

PHx was performed in wild-type (WT) and intestinal *Sirt1* KO mice (SIRT^intKO^), and samples were harvested at different timepoints after surgery. Liver damage was assessed by analysis of serum transaminases, showing significantly higher levels of alanine aminotransferase (ALT) and aspartate aminotransferase (AST) in SIRT^intKO^ mice at 24 hours after PHx compared with WT littermates (Figure 1*A*). Histopathologic analysis on tissue sections confirmed extensive liver parenchymal damage in SIRT^intKO^ mice, with profuse areas of necrosis at 24 hours after PHx compared with WT mice (Figure 1*B*).

The regeneration of the liver after PHx is mainly mediated by the replication of hepatocytes that proliferate during the initial 24 to 48 hours after surgery.^1^^,^^15^ To determine the impact of intestinal-specific deletion of SIRT1 on pathways associated with hepatocyte proliferation after PHx, we performed Western blot analysis of proteins related to cell-cycle progression. Our results revealed a reduction in the phosphorylation of extracellular signal-regulated kinase (ERK)1 and ERK2 in SIRT^intKO^ mice compared with WT mice (Figure 1*C*), indicating impaired G1 phase progression.^16^ Further analysis of liver tissues showed reduced expression of cyclin D1 in liver samples from SIRT^intKO^ mice compared with WT mice throughout the proliferative phase of liver regeneration (Figure 1*D*).

To further assess hepatocyte proliferation, we performed immunohistochemical analysis using an anti-bromodeoxyuridine (BrdU) antibody in liver tissue samples from mice receiving the nucleotide analogue BrdU before culling. Our results indicated an apparent reduction in the percentage of BrdU-positive hepatocytes in SIRT^intKO^ mice at 40 hours after PHx that was significant at 48 hours compared with WT littermates (Figure 1*E* and *F*).

Finally, we assessed hepatocyte mitosis in liver sections and found a significant reduction in phosphorylated histone H3 (p-histone H3)-positive hepatocytes in SIRT^intKO^ mice at 48 hours after PHx compared with WT mice (Figure 1*G* and *H*), supporting the reduced replicative capacity of hepatocytes in KO mice.

Together, our results show that the intestinal specific depletion of SIRT1 has a detrimental impact in the regenerative response of the liver, leading to profuse necrotic cell death at 24 hours after PHx and a reduced proliferative response in hepatocytes at 40 and 48 hours after surgery compared with WT littermates.

### Intestinal Sirtuin 1 Deletion Leads to Changes in the Transcriptomic Profiling and Attenuated Signal Transducer and Activator of Transcription 3 Phosphorylation in the Liver During the Priming Phase After Partial Hepatectomy

**Figure 2:**
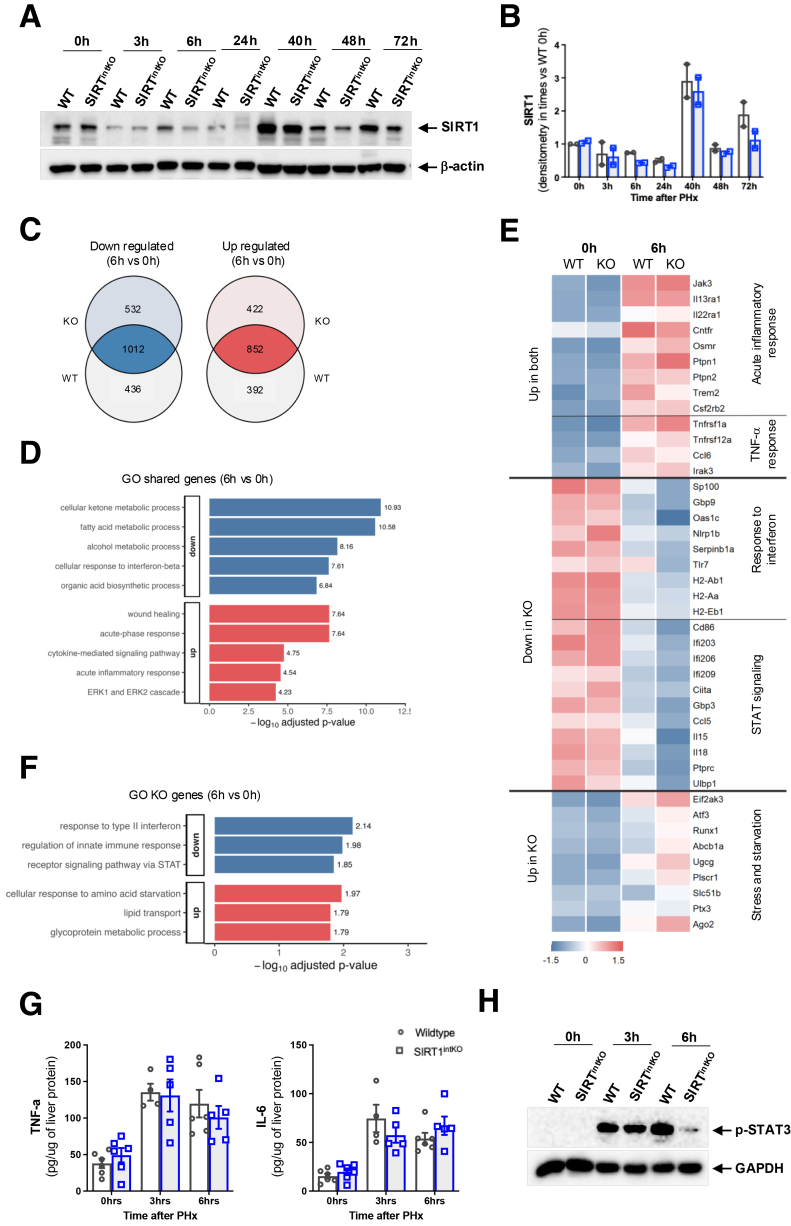
**Intestinal-specific SIRT1 deletion alters transcriptomic profiling and STAT signaling during the priming phase after PHx.** (*A*) Western blot analysis of liver tissue samples showing SIRT1 expression in SIRT^intKO^ and WT mice at 0, 3, 6, 24, 40, 48, and 72 hours after PHx and (*B*) densitometry of SIRT1 protein levels across the indicated time points. (*C*) Venn diagram showing DEGs shared or specific to WT and SIRT^intKO^ livers at 6 hours post-PHx (n = 5 per group). (*D*) GO enrichment analysis of shared DEGs in (*A*). (*E*) Heatmap showing scaled expression (z-score) of representative genes from enriched GO terms in (*D*). (*F*) GO enrichment analysis of SIRT^intKO^-specific DEGs (up- and downregulated), performed as in (*D*). (*G*) TNF-α and IL6 protein expression quantified by enzyme-linked immunosorbent assay in whole protein liver extracts at 0, 3, and 6 hours after PHx. (*H*) Western blot analysis of liver tissue samples showing attenuated p-STAT3 expression in SIRT^intKO^ vs WT mice at 3 and 6 hours after PHx, Analyses were done from n = 5–6 mice. Values are mean ± standard error of the mean. Statistical differences were determined using 2-way analysis of variance with Holm–Sidak correction. For transcriptomics, statistical differences were determined using DESeq2, *P*- and q-value cutoffs = .05 using Benjamini–Hochberg correction and |log_2_ fold change| > 1.

In previous work, we described that the overexpression of SIRT1 had a detrimental impact on liver regeneration.^14^ To determine whether intestinal SIRT1 deletion influenced liver SIRT1 expression, we performed immunoblotting on liver whole protein extract followed by densitometry. Our results confirmed our previous observations that SIRT1 expression is reduced at initial stages after PHx (6 hours, 24 hours) to later increase at 40 to 72 hours after PHx in WT mice. We found that liver SIRT1 expression was marginally lower at 6 hours and 24 hours after PHx in SIRT^intKO^ mice compared with WT mice, although these differences did not reach statistical significance (Figure 2*A* and *B*).

To further investigate the potential causes of the reduced hepatocyte proliferation in SIRT^intKO^ mice after PHx, we focused initially on the priming phase, where progression from the G0 to the G1 phase of the cell cycle occurs within the first 6 hours after PHx in mice.

To characterize the molecular programs underlying the differential responses observed in SIRT^intKO^ mice compared with their WT littermates during the priming phase, we performed bulk RNA sequencing (RNA-seq) on liver samples at 0 and 6 hours after PHx in both genotypes. Differentially expressed genes (DEGs) with the same direction of change in WT and SIRT^intKO^ mice were defined as shared DEGs. Genes differentially expressed only in SIRT^intKO^ mice were defined as SIRT^intKO^-specific responses.

At 6 hours after PHx, transcriptional changes were largely shared between genotypes. Over 50% of DEGs overlapped between WT and SIRT^intKO^ mice (Figure 2*C*). Shared downregulated genes were enriched for metabolic processes, including fatty acid, ketone, alcohol, and organic acid metabolism (Figure 2*D*; Supplementary Table 1). These included key regulators such as *Srebf1, Ppard, Mlxipl*, and multiple members of the *Ces* (lipid homeostasis), *Cyp* (detoxification and hormone metabolism), and *Akr* (reduction of various carbonyl-containing compounds) gene families (Supplementary Table 1), suggesting a coordinated suppression of hepatic metabolic activity in both genotypes at 6 hours after PHx. Shared upregulated genes at 6 hours were enriched in pathways related to inflammation and acute-phase response (Figure 2*B* and *C*), involving genes such as members of the Janus kinase/signal transducer and activator of transcription (STAT) signaling pathway (*Jak3*, *Osmr*) and interleukin receptors (*Il13ra1*, *Il22ra1*), reflecting activation of cytokine signaling. Induction of *Tnfrsf1a*, *Tnfrsf12a*, *Csf2rb2*, and the chemokine *Ccl6*, indicating engagement of canonical tumor necrosis factor (TNF)-mediated inflammatory pathways were also upregulated in both WT and SIRT^intKO^ mice (Figure 2*D* and *E*). Together, these data reflect that the early inflammatory and tissue repair responses typical of liver regeneration are similarly regulated in WT and SIRT^intKO^ mice at 6 hours after PHx.

Despite the overall similarity, we detected SIRT^intKO^-specific responses that included a significant downregulation in genes involved in STAT signaling (*Cd86*, *Ciita*, and the chemokines *Ccl5* and *Il15*) and regulation of immune response, characterized by responses to interferon-stimulated genes (*Ifi* and *Gbp* family members, *Oas1c*, *Sp100*), as well as components of the antigen presentation machinery (*H2-Ab1*, *H2-Aa*, *H2-Eb1*) and innate immune receptors as well as inflammasome-related genes (*Tlr7*, *Nlrp1b*) (Figure 2*E* and *F*).

In turn, genes linked to stress and starvation responses, including *Atf3* and *Eif2ak3* (also known as protein kinase RNA-like endoplasmic reticulum [ER] kinase) were upregulated in SIRT^intKO^ mice, which was not observed in WT animals (Figure 2*E* and *F*).

Hepatocyte proliferation is primed by the secretion of TNF-α and interleukin6 (IL6) from neighboring Kupffer cells that activate signaling cascades in hepatocytes, including phosphorylated STAT 3 (p-STAT3), to promote cell cycle progression.^17^ In line with the transcriptomic analysis (Figure 2*D* and *E*), our results show that, although TNF-α and IL6 protein levels are not significantly different in WT compared with SIRT^intKO^ mice at 3 and 6 hours after PHx (Figure 2*G*), we found an evident reduction in p-STAT3 levels in SIRT^intKO^ mice, particularly at 6 hours after surgery (Figure 2*H*), and also, accordingly, to the downregulation of the STAT pathway as indicated by the Gene Ontology (GO) analysis (Figure 2*E* and *F*).

Together, these results point to a modest early divergence in how SIRT^intKO^ mice livers respond to PHx-induced stress, mainly concerning STAT and cell metabolism responses, which might set the stage for the attenuated proliferation observed at later time points.

### Transcriptomic Profiling Reveals Dynamic and Genotype-Specific Changes in SIRT^intKO^ Mice Compared With Wild-Type Mice During Liver Regeneration at 24 Hours After Partial Hepatectomy

To further understand the mechanisms mediating the detrimental phenotype of SIRT^intKO^ after PHx, we next performed comparative analysis of bulk RNA-seq datasets obtained from liver samples from WT and SIRT^intKO^ mice at 0 and 24 hours after PHx.

**Figure 3:**
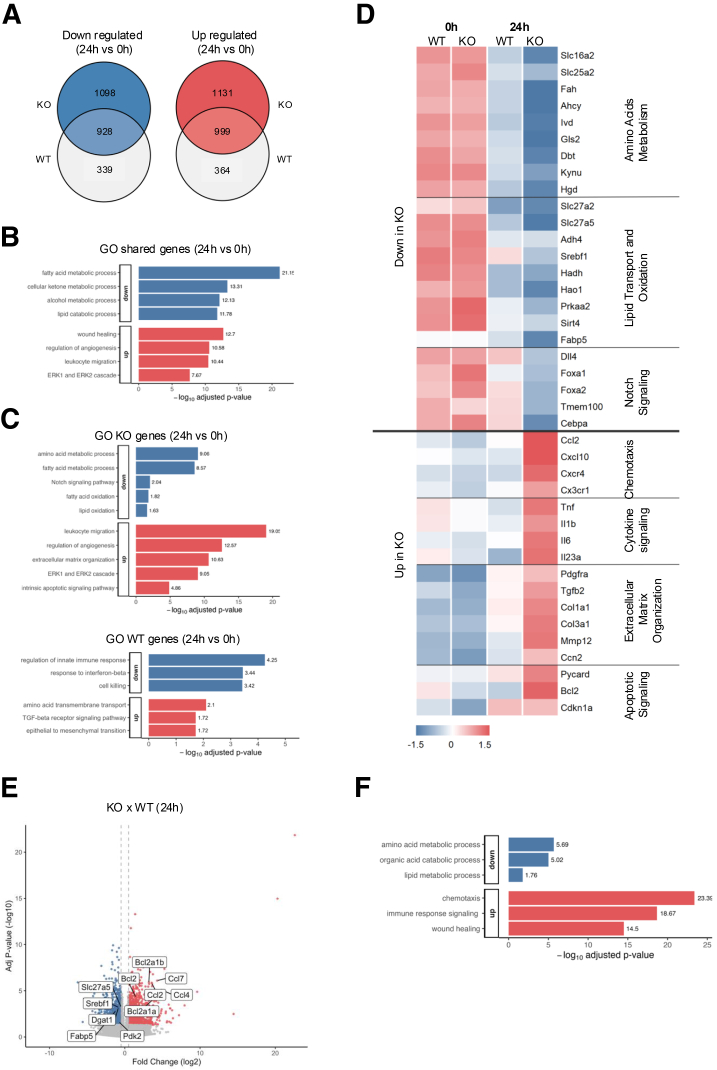
**Transcriptional profiling of livers from WT compared with intestinal-specific SIRT1 KO mice at 24 hours after PHx.** (*A*) Venn diagram showing DEGs shared or specific to WT (n = 5) and SIRT^intKO^ (n = 7) livers at 24 hours post-PHx. (*B*) GO enrichment analysis of shared DEGs from (*A*). (*C*) GO enrichment analysis of genotype-specific DEGs in WT and SIRT^intKO^ (up- and downregulated), performed as in (*B*). (*D*) Heatmap showing scaled expression (z-score) of selected genes from enriched GO terms in (*B*) and (*C*). (*E*) Volcano plot showing DEGs between SIRT^intKO^ and WT mice at 24 hours post-PHx. *Red*, genes upregulated in KO; *blue*, genes downregulated in KO; *gray*, not significant. (*F*) GO enrichment analysis of shared DEGs from (*E*). For transcriptomics, statistical differences were determined using DESeq2, *P*- and q-value cutoffs = .05 using Benjamini–Hochberg correction and |log_2_ fold change| > 1.

As for the 6-hour timepoint, we first identified DEGs within each genotype by comparing 24-hour vs 0-hour samples. We then defined shared DEGs as those significantly modulated in both genotypes with the same direction of change and SIRT^intKO^-specific DEGs as those significantly altered only in the SIRT^intKO^ group. Transcriptional differences between WT and SIRT^intKO^ mice became more pronounced than in the 6-hour timepoint (Figure 3*A*; Supplementary Table 1), although a core regenerative program was still shared, with downregulated pathways involving ketone metabolism and lipid catabolism and upregulated genes centered around wound healing, leukocyte migration, and angiogenesis (Figure 3*B*; Supplementary Table 1), SIRT^intKO^ livers displayed a broader set of unique DEGs (Figure 3*A*, *C–E*).

In SIRT^intKO^ mice, key genes involved in amino acid (*Gls2, Kynu*), fatty acid (*Hadh, Hao1, Slc27a2, Slc27a5, Fabp5*), and lipid metabolism (*Srebf1, Slc16a2*) were specifically downregulated at 24 hours after PHx (Figure 3*C* and *D*). The early stress and starvation response observed at 6 hours (Figure 2*C* and *D*) may underlie this impaired metabolic profile, suggesting that inadequate early adaptation compromises hepatic metabolic reactivation during regeneration. We also observed reduced expression of the transcription factor *Foxa2*. Foxa2 has been described to regulate BA homeostasis and ER stress in mice and to negatively regulate multidrug resistance protein (MDR) 1,^18^ the BA transporter whose expression we found increased in SIRT^intKO^ mice (Figure 2*E*).

Specific upregulated genes in SIRT^intKO^ mice were enriched in proinflammatory and tissue damage-associated pathways (Figure 3*C* and *D*). Notably, leukocyte migration and chemotaxis involved several chemokines (*Ccl2, Cxcl10, Ccl3, Ccl4, Ccl6, Ccl7, Ccl11, Ccl12*) and their receptors (*Cxcr2, Cxcr4, Cx3cr1*), indicative of robust recruitment of immune cells in SIRT^intKO^ mice (Figure 3*C* and *D*; Supplementary Table 1). Genes involved in extracellular matrix organization, including collagens (*Col1a1, Col1a2, Col3a1, Col4a2, Col5a1, Col5a2*), *Mmp12, Pdgfra, Loxl1*, and matricellular proteins (*Ccn1, Ccn2, Adamts2, Adamts9*), were strongly upregulated in SIRT^intKO^ mice, reflecting ongoing tissue remodeling (Figure 3*C* and *D*; Supplementary Table 1). Additionally, genes relevant to intrinsic apoptotic regulators such as *Bcl2* and *Pycard* (ASC) were upregulated in SIRT^intKO^ mice. Interestingly, *Cdkn1a* (p21) was also increased in SIRT^intKO^ compared with WT mice, pointing to an increased senescence in KO animals at 24 hours after PHx (Figure 3*C* and *D*).

In WT livers, the upregulation of amino acid metabolism was accompanied by activation of transforming growth factor-β and epithelial to mesenchymal-related pathways (Figure 3*C*), supporting a coordinated metabolic and remodeling response that may align with a proper tissue repair. In contrast, SIRT^intKO^ livers failed to activate these programs and instead showed sustained proinflammatory and damage-associated gene expression.

To further characterize the metabolic alterations in SIRT^intKO^ livers at 24 hours after PHx, we directly compared WT and KO transcriptomes at this timepoint. This analysis revealed significant enrichment of pathways related to lipid, fatty acid, amino acid, and energy metabolism, including fatty acid oxidation, organic acid catabolic and biosynthetic processes, and generation of precursor metabolites and energy. Among the most significantly upregulated genes in WT compared with KO were *Dgat1* (triglyceride synthesis), *Slc27a5* (fatty acid transport), *Srebf1* (lipogenesis), *Star* (cholesterol trafficking), and *Fabp5* (fatty acid binding) (Figure 3*E* and *F*; Supplementary Table 1. These findings indicate that WT livers engage robust metabolic reprogramming at 24 hours to support regeneration, whereas this response is blunted in SIRT^intKO^ mice.

Together, these results suggest that, although initial regenerative cues are broadly conserved, intestinal SIRT1 regulates the reactivation of hepatic metabolism, essential for providing the energy required to support hepatocyte proliferation during liver regeneration. In the absence of intestinal SIRT1, the liver remains locked in a proinflammatory state, likely responding to the extensive parenchymal damage associated with increased BA-induced hepatotoxicity, overall failing to initiate proper tissue recovery.

### Intestinal Sirtuin 1 Deletion Leads to Increased Hepatocyte Senescence and Progenitor Cells Proliferation After Partial Hepatectomy

**Figure 4:**
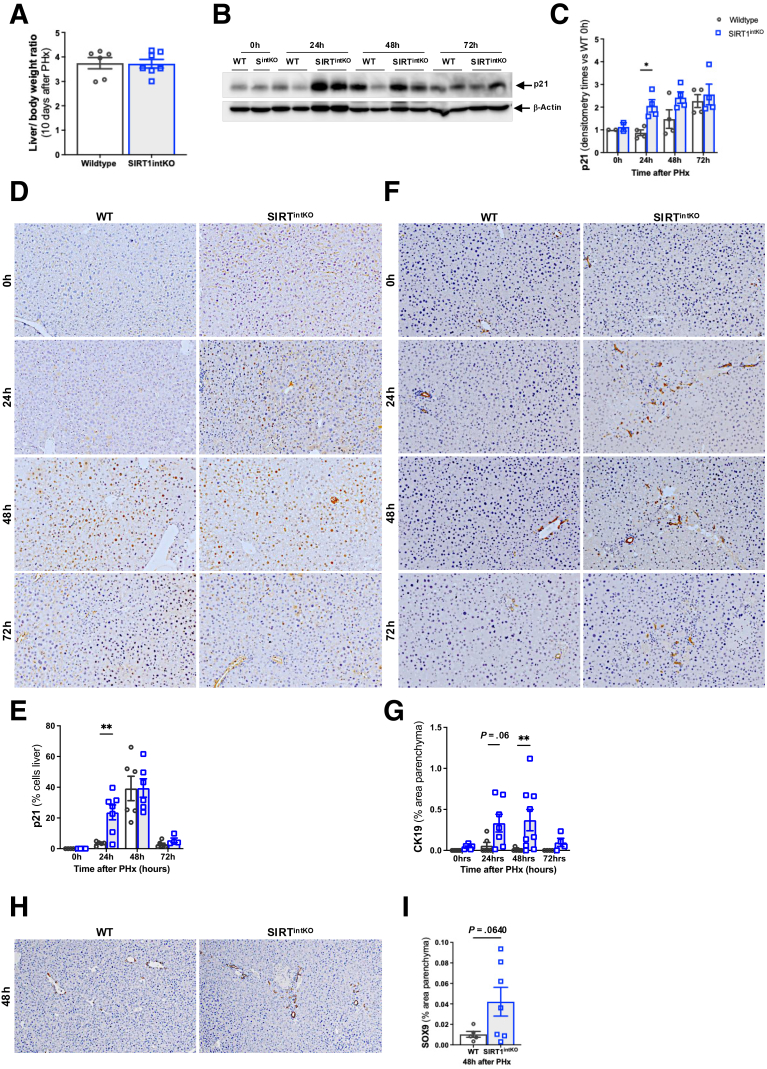
**Restoration of liver mass in intestinal-specific SIRT1 KO mice is associated with early senescence and oval cell activation after PHx.** (*A*) Liver weight/body weight ratio in WT vs SIRT^intKO^ mice at 10 days after PHx. (*B*) Western blot analysis of liver tissue samples showing p21 expression in SIRT^intKO^ and WT mice at 0, 24, 48, and 72 hours after PHx and (*C*) densitometry of p21 protein levels across the indicated time points. (*D*) IHC showing p21-positive hepatocytes and (*E*) further quantification showing the percentage of p21-positive cells ppf. (*F*) IHC showing positive CK19 staining in cells and (*G*) quantification of the percentage of CK19-positive area within the parenchyma (but not in bile duct areas) at 0, 24, 48, and 72 hours after PHx in WT vs SIRT^intKO^ mice. (*H*) IHC showing positive SOX9 staining within the parenchyma and (*I*) quantification of the percentage of SOX9 positive per area at 48 hours after PHx in WT vs SIRT^intKO^ mice. Analyses were done from n = 5–9 mice. Representative images are shown from 10× magnification. Values are mean ± standard error of the mean. Statistical differences were determined using 2-way analysis of variance with Holm–Sidak correction. ∗*P* < .05, ∗∗*P* < .01.

Restoration of liver mass is achieved by day 7 to 10 after PHx in mice.^1^ Interestingly, despite impaired hepatocyte replication, evidenced by lower proliferation and mitosis (Figure 1), liver mass was restored in SIRT^intKO^ mice at comparable levels than in WT littermates at 10 days after PHx (Figure 4*A*), pointing to the overall successful regeneration of the liver in mice lacking intestinal SIRT1.

Senescence is a cellular response to stress and damage leading to cell-cycle arrest, driven by the expression of p21 (*Cdkn1a*) a well-accepted marker of senescence.^19^ During liver regeneration, hepatocyte senescence is activated at later phases of the cell cycle as an essential part of the termination phase, to halt hepatocyte proliferation and avoiding liver overgrowth, with p21 expression reaching its maximum at 48 hours.^20^

Accordingly, with the reduced hepatocyte proliferation we observed in SIRT^intKO^ mice and following our results from transcriptional analyses, showing upregulation of p21 expression (Figure 3*D*), we found a significant increase in p21 protein expression in livers from SIRT^intKO^ mice compared with WT mice at 24 hours after PHx by immunoblotting and further densitometry (Figure 4*B* and *C*). p21 protein levels were further increased in WT mice at 48 hours after PHx and remained elevated in SIRT^intKO^ mice. These results were confirmed by immunohistochemistry (IHC) using an anti-p21 antibody in liver tissue sections that showed a significant increase in the percentage of p21-positive hepatocytes in SIRT^intKO^ mice compared with WT mice at 24 hours after PHx (Figure 4*D* and *E*). The protein expression and number of p21-positive hepatocytes remained elevated at 48 hours in livers from SIRT^intKO^ mice, then decreased at 72 hours after surgery (Figure 4*B–E*). In WT mice, the presence of p21-positive hepatocytes was not detected at 24 hours but reached its expected peak at 48 hours after PHx, then was reduced at 72 hours after surgery (Figure 4*B–E*).

When hepatocyte proliferation is impaired, liver regeneration can occur from the proliferation and differentiation of progenitor cells,^21^ precursors of liver progenitor oval cells that express biliary markers,^22^ including cytokeratin 19 (CK19), as described by Roskams et al.^23^ To determine whether restoration of liver mass in SIRT^intKO^ mice involved oval cell activation, we performed IHC analysis of CK19 expression on liver sections. Accordingly, we found the significant presence of CK19-positive cells, with a morphology concordant with oval cells, throughout the parenchyma of SIRT^intKO^ mice at 24 and 48 hours after PHx compared with their WT littermates, where CK19-positive cells were almost not detected in the parenchyma but were restricted to the bile duct areas (Figure 4*F* and *G*). These observations were confirmed by immunostaining of liver sections using SRY-box transcription factor 9 (SOX9), a marker for pluripotent stem cells. We detected increased presence of SOX9-positive cells within the liver parenchyma of SIRT^intKO^ mice at 48 hours after PHx, whereas SOX9-positive staining remained restricted to the bile ducts in WT liver samples (Figure 4*H* and *I*).

Together, these results support that the intestinal deletion of SIRT1 leads to increased early senescence of hepatocytes and activation of the hepatic progenitor cell compartment as a compensatory response to regenerate the liver mass after PHx in SIRT^intKO^ mice.

### Intestinal Sirtuin 1 Deletion Leads to Bile Acid Accumulation in the Liver During Regeneration, Which Positively Associates With Increased Liver Damage After Partial Hepatectomy

Next, we aimed to better understand the mechanism mediating the detrimental impact of intestinal SIRT1 deletion during liver regeneration.

**Figure 5:**
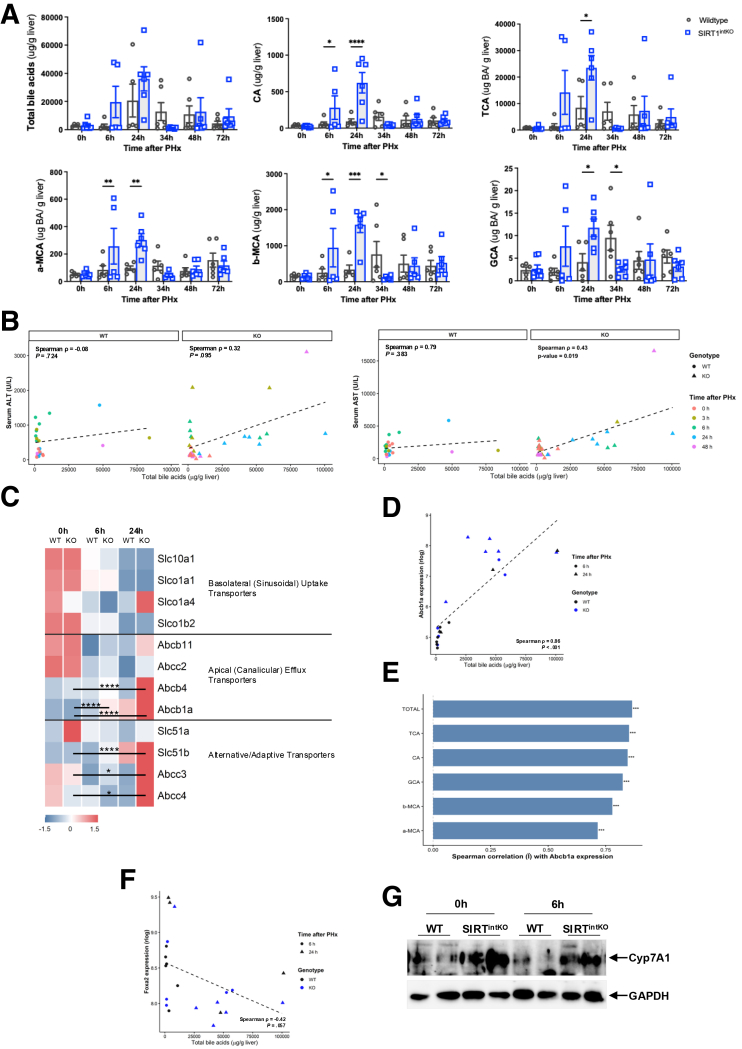
**Intestinal-specific SIRT1 deletion leads to reduced FXR-FGF15-FGFR4 activation after PHx.** (*A*) Quantification of the total liver BA pool and specific BA species by liquid chromatography-mass spectrometry. (*B*) Correlations between total BA (μg/g liver) and ALT (*left panels*; U/L) or AST (*right panels*; U/L), stratified by genotype. (*C*) Heatmap showing scaled expression (z-score) of BA transporters. Differential expression was assessed within each genotype. Genes similarly regulated in both genotypes were not highlighted. (*D*) Correlation between hepatic *Abcb1a* expression and total BA (μg/g liver). (*E*) Correlation between hepatic *Abcb1a* expression and individual measured BAs. (*F*) Correlation between hepatic *Foxa2* expression and total BA (μg/g liver). (*G*) Western blot analysis of liver tissue samples showing differential CYP7A1 expression in WT vs SIRT^intKO^ mice at 6 hours after PHx. Analyses were done from n = 4–9 mice. Values are mean ± standard error of the mean. Statistical differences were determined using 2-way analysis of variance with Holm–Sidak correction. Correlations were assessed using Spearman’s rank correlation. *P* < .05, ∗∗*P* < .01, ∗∗∗*P* <.001, ∗∗∗∗*P* < .001 (WT vs SIRT^intKO^ after PHx). For transcriptomics, statistical differences were determined using DESeq2. CA, cholic acid; TCA, taurocholic acid.

Although BAs are essential for liver regeneration,^2^ an excess of BAs in the liver is toxic, causing hepatocellular death.^24^ Our results showed a tendency for increased total BA accumulation in the livers of SIRT^intKO^ mice compared with WT mice at 6 and 24 hours after PHx, although this did not reach statistical significance. At later timepoints, BA levels decreased both in WT and SIRT^intKO^ mice (Figure 5*A*). Still, we detected a significantly higher presence of cholic acid (CA), α-muricholic acid (MCA), β-MCA, taurocholic acid, and glycocholic acid (GCA) in SIRT^intKO^ mice at 24 hours after PHx (Figure 5*A*). These results supported the accumulation of BAs as probable mediators of the profuse liver injury, including wide areas of necrosis, we observed in SIRT^intKO^ mice at 24 hours after PHx (Figure 1*A* and *B*).

To confirm this, we analyzed correlations between total liver BAs and serum ALT and AST levels. In SIRT^intKO^ mice, total BAs showed a borderline positive association with ALT (*P* = .092; rho = 0.32) and a significant positive correlation with AST (*P* = .019; rho = 0.43). In contrast, WT mice showed no significant correlation between BAs and either transaminase (Figure 5*B*). These findings suggest that the relationship between increased BA levels and liver injury markers is more prominent in the context of intestinal SIRT1 deletion.

Accumulation of BAs in the liver may result from disruption of BA transport, including alterations in influx and efflux pathways. Analysis of our RNA-seq data at basal conditions (0 hours) and at 6 hours and 24 hours after PHx revealed no significant differences in the expression of basolateral (sinusoidal) BA uptake transporters across timepoints in either genotype (Figure 5*C*). Similarly, the expression of the canalicular transporter *Abcb11* (bile salt export pump) was not significantly altered in SIRT^intKO^ mice. In contrast, the expression of several apical (canalicular) efflux transporters and alternative/adaptive BA transporters increased over time, specifically in SIRT^intKO^ mice following PHx. Notably, *Abcb4* (MDR2), *Abcb1a* (MDR1), *Slc51b* (organic solute transporter β), *Abcc3* (multidrug resistance-associated protein [MRP]3), and *Abcc4* (MRP4) showed increased expression across timepoints in SIRT^intKO^ mice, whereas no significant changes were observed in WT mice. In the SIRT^intKO^ mice, MDR1 was already elevated at 6 hours after PHx (Figure 2*E*) and remained increased at 24 hours.

The increased expression of these efflux and alternative transporters likely reflects a compensatory response to the elevated BA levels observed in the liver of SIRT^intKO^ mice. Indeed, Spearman correlation analysis revealed a strong positive correlation between MDR1 expression and total hepatic BA (*P* < 2.2 × 10^−16^; rho = 0.85) (Figure 5*D*) after PHx. Notably, mice presenting the highest BA levels also exhibited the highest MDR1 expression. Consistently, several individual BAs as well as the total BA pool showed significant correlations with MDR1 expression in SIRT^intKO^ mice at 6 and 24 hours after PHx (Figure 5*E*).

Interestingly, our RNA-seq data analysis showed decreased expression of *Foxa2* (Figure 3*E*), a transcription factor known to regulate BA homeostasis,^18^ which we confirmed to negatively correlate with BA levels in the liver after PHx (Figure 5*F*).

Together, our results showing decreased Foxa2 and increased MDR1 expression, which correlate with strong accumulation of BAs, support the pronounced disruption in BA metabolism in SIRT^intKO^ mice at 24 hours after PHx.

We next explored BA synthesis as a potential contributor to the accumulation of BAs in the liver during regeneration. Liver BA metabolism is tightly controlled by the downregulation of *Cyp7a1*, which responds to intestinal FXR signaling, and inhibits BA synthesis in the liver.^25^ Analysis of CYP7A1 expression, the main activator of BA synthesis, by immunoblotting, showed an apparent increase in the protein levels in SIRT^intKO^ mice basally and at 6 hours after PHx compared to WT mice (Figure 5*G*).

### Intestinal Sirtuin 1 Deletion Impairs Intestinal FXR–FGF15 Activation After Partial Hepatectomy, Whereas Intestinal FXR Activation Restores Liver Regeneration Despite Sirtuin 1 Deficiency

Activation of intestinal FXR regulates liver BA metabolism via the induction of intestinal FGF15, which promotes activation of FGFR4 in the liver and the consequent downregulation of CYP7A1.^25^ Based on the work of others and our previous work showing that SIRT1 is an upstream regulator of FXR activation,^9^^,^^11^ we anticipated that the deletion of intestinal SIRT1 may affect FXR signaling in the intestine during liver regeneration.

**Figure 6:**
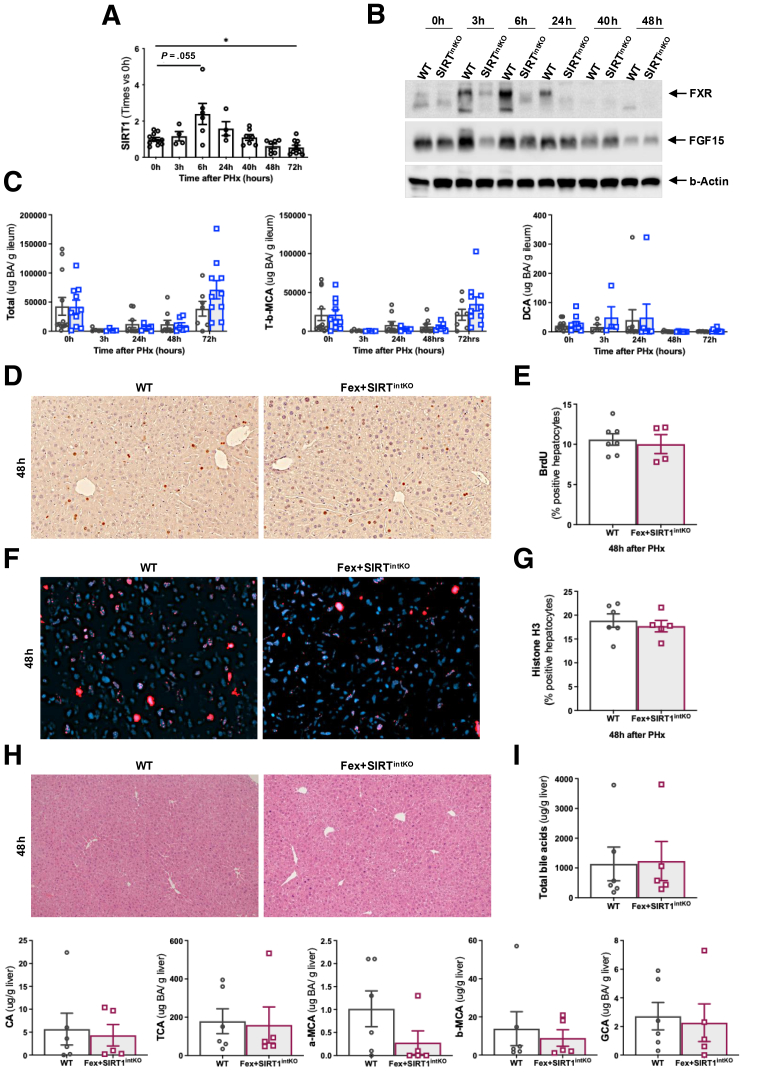
**Intestinal SIRT depletion leads to disruption of the FXR-FGF15 axis. Activation of intestinal FXR signaling with Fex restores hepatocyte proliferation in SIRT^intKO^ mice after PHx.** (*A*) Relative Sirt1 messenger RNA expression in small intestine scraps of WT mice at the indicated time points after PHx. (*B*) Western blot analysis of small intestine scraps showing differential FXR and FGF15 expressions in WT vs SIRT^intKO^ mice at different timepoints after PHx. (*C*) Quantification of the total intestine BA pool and specific BA species in small intestine contents by liquid chromatography-mass spectrometry. (*D*) IHC showing BrdU-positive hepatocytes and (*E*) quantification of the percentage of positive cells at 48 hours after PHx in WT vs Fex-treated SIRT^intKO^ (Fex+SIRT^intKO^) mice. (*F*) Immunofluorescence showing p-HistoneH3–positive cells and (*G*) quantification of the percentage of positive cells at 48 hours after PHx in WT vs Fex+SIRT^intKO^ mice. (*H*) Hematoxylin and eosin staining of liver sections from WT vs Fex+SIRT^intKO^ at 48 hours after PHx. (*I*) Quantification of total BA levels in WT and Fex+SIRT^intKO^ mice at 48 hours after PHx. Analyses were done from n = 4–6 mice. Representative images are shown from 10× (*D* and *H*) and 20× (*F*) magnification. Values are mean ± standard error of the mean. Statistical differences were determined using 2-way analysis of variance with Holm–Sidak correction. ∗*P* < .05. CA, cholic acid; DCA, deoxycholic acid; TCA, taurocholic acid.

Initially, we analyzed the gene expression of SIRT1 in the small intestine after PHx and found an early upregulation at 6 hours after PHx that was gradually reduced from 24 to 72 hours after liver resection in WT mice (Figure 6*A*).

Further analysis of small intestinal epithelial cell samples showed that FXR expression increased at early timepoints, mainly 3 and 6 hours after PHx in WT mice, whereas its expression remained low in SIRT^intKO^ mice after surgery (Figure 6*B*). In accordance with the regulation of FXR, we observed increased expression of FGF15 in WT mice at 3 and 6 hours after PHx, whereas this upregulation was not observed in SIRT^intKO^ mice (Figure 6*B*).

BAs are the main activators of intestinal FXR signaling, whereas conjugated BAs like T-β-MCA can act as antagonists of this nuclear factor.^26^ Analysis of the total pool of BAs in the small intestine of WT and SIRT^intKO^ mice after PHx showed a sharp decrease after surgery that recovered pre-PHx levels only after 72 hours both in WT and SIRT^intKO^ mice at similar levels (Figure 6*C*). A more detailed analysis of the BA pool composition confirmed that no significant differences were observed in T-α-MCA (data not shown) or T-β-MCA. Secondary BAs, including deoxycholic acid, were also not significantly different between WT and SIRT^intKO^ mice (Figure 6*C*).

Together, these results show that intestinal-specific SIRT1 deletion negatively impacts the activation of the FXR/FGF15 axis in the small intestine during liver regeneration, influencing BA metabolism and contributing to the accumulation of toxic BAs after a PHx.

To determine that FXR/FGF15 activation in the small intestine during liver regeneration is downstream of SIRT1, we treated SIRT^intKO^ mice with fexaramine (Fex), an intestinal-restricted FXR agonist that we and others have described to have beneficial effects in restoring intestinal and liver function.^27^^,^^28^

We found that treatment with Fex (1 hour before surgery) successfully restored hepatocyte proliferation, as evidenced by BrdU staining on liver sections that showed a comparable percentage of positive cells in WT vs Fex/SIRT^intKO^ mice at 48 hours after PHx (Figure 6*D* and *E*). In line with this, p-histone H3 immunostaining confirmed the restored regenerative capacity in Fex/SIRT^intKO^ mice after PHx that showed comparable numbers of mitotic cells in the liver (Figure 6*F* and *G*). Histopathologic analysis of livers stained with hematoxylin and eosin confirmed the lack of parenchymal damage in WT and Fex/SIRT^intKO^ mice at 48 hours after surgery (Figure 6*H*). The absence of obvious cellular damage observed is associated with comparable total BA levels in WT and Fex/SIRT^intKO^ mice at 48 hours after PHx (Figure 6*I*). Detailed analysis of the different BA species confirmed the comparable levels of cholic acid, taurocholic acid, α-MCA/β-MCA, and GCA (Figure 6*I*) in Fex/SIRT^intKO^ mice compared with WT mice 48 hours after surgery.

Together, our results show that intestinal-specific activation of FXR restores hepatocyte proliferation in SIRT^intKO^ mice, supporting that intestinal SIRT1 is an upstream regulator of FXR during liver regeneration.

## Discussion

Here, we demonstrate that intestinal SIRT1 controls liver regeneration by acting as an upstream regulator of FXR/FGF15 signaling, preventing liver parenchymal damage and regulating BA metabolism and hepatocyte proliferation after PHx in mice.

BAs are essential for liver regeneration,^2^ whereas the accumulation of toxic BAs contributes to parenchymal liver damage.^24^

We found profuse parenchymal liver damage in SIRT^intKO^ mice within the first 24 hours after PHx, with significantly elevated necrosis and serum transaminase levels that positively correlated with BA accumulation at 6 and 24 hours after surgery in KO mice compared with WT mice.

Although we did not detect significant differences in the expression of basolateral transporters responsible for BA uptake into hepatocytes, we observed increased expression of apical efflux pumps and alternative transporters in SIRT^intKO^ mice within the first 24 hours after PHx. These changes likely reflected the elevated presence of BAs rather than contributed to their accumulation in the liver. Interestingly, we found an upregulation of MDR1, an ATP-dependent canalicular efflux pump^29^^,^^30^ that was described to be highly expressed during primary biliary cholangitis and to positively correlate with liver necrosis in severe human disease.^29^ Previous work showed that the transcription factor forkhead box A2 controls BA homeostasis by regulating MDR1 and other transporters (MRP2, MRP4) in the liver, independently of FXR–small heterodimer partner signaling.^18^ Similar to these findings by Bochkis et al, we found that decreased forkhead box A2 expression in SIRT^intKO^ mice after PHx was associated with increased MDR1, which significantly correlated with the increased accumulation of BA in the livers of these KO mice. Still, our results showing that other relevant BA transporters remained unaffected, point to additional alterations in BA metabolism occurring in SIRT1 intestinal-deficient mice.

FXR is the main regulator of BA metabolism, as its activation in the small intestine epithelial cells (enterocytes) induces the upregulation of FGF15/19, which inhibits BA synthesis in the liver by repressing CYP7A1 via FGFR4 signaling.^4^^,^^25^^,^^31^ We and others have described that SIRT1 acts as an upstream regulator of FXR in the liver,^9^^,^^11^ and previous work using SIRT^intKO^ established that intestinal SIRT1 controls systemic BA metabolism by promoting FXR/FGF15 activation during homeostasis.^12^ Together, this evidence allowed us to propose that the depletion of SIRT1 in the intestine may alter the gut–liver BA metabolism via disruption of FXR/FGF15 signaling, overall affecting liver regeneration.

Our results showed that intestinal SIRT1 depletion led to disruption of the FXR-FGF15 axis, with reduced intestinal FXR and FGF15 expression at 6 and 24 hours after PHx, which was associated with increased CYP7A1 expression in the liver, supporting the BA accumulation observed in SIRT^intKO^ mice.

Beyond regulating BA metabolism, the role of FXR in controlling liver regeneration was established using constitutive FXR KO mice that showed impaired hepatocyte proliferation after PHx.^2^ Further work by Zhang and colleagues using cell-specific KO mice demonstrated that both hepatocyte- and intestinal epithelial cell–FXR activation are relevant to regulating liver regeneration while using different mechanisms. Importantly, that study demonstrated the role of intestinal FXR in activating FGF15.^5^ Further studies supported that FGF15 is essential to mediate hepatocyte proliferation and liver regeneration,^32^ whereas the deletion of FGF15 is associated with accumulation of BAs in the liver after PHx.^3^^,^^7^ Our results showing attenuation of hepatocyte proliferation in SIRT^intKO^ mice alongside with reduced FXR/FGF15 activation are in accordance with the previously described role of these factors in controlling hepatocyte proliferation^2^^,^^3^^,^^5^^,^^7^ and point to SIRT1 as an upstream regulator of the FXR/FGF15 axis in the small intestine during liver regeneration.

Hepatocyte proliferation during liver regeneration is primed by cytokines including TNFα and IL6 secreted from Kupffer cells that activate signaling cascades in hepatocytes, including STAT3, allowing cell-cycle progression from G0 to G1 phase and further cell proliferation.^15^^,^^17^ Here, we found reduced expression of STAT pathway genes and reduced activation of STAT3 signaling at 6 hours after PHx in SIRT^intKO^ mice compared with WT mice, whereas TNF and IL6 protein expression levels were comparable. These results suggest that intestinal deletion of SIRT1 does not affect Kupffer cell activation after PHx, whereas the reduced phosphorylation of STAT3 could be mediated by the excess of BA in the liver resulting from disruption of FXR/FGF15 signaling. Similar observations were described by Kong and colleagues in a study where FGF15^-/-^ mice led to the accumulation of BAs in the liver that was associated with reduced STAT3 phosphorylation, impaired hepatocyte proliferation, and increased parenchymal damage after PHx.^3^ Interestingly, although transcriptional changes were generally comparable between WT and SIRT^intKO^ mice at 6 hours after PHx, GO analysis highlighted features of initiation of cell death, metabolic stress, and ER stress in SIRT^intKO^ mice, with increased expression of Atf3 that influences the shift to necroptosis in cells^33^ and Eif2ak3/protein kinase RNA-like ER kinase involved in ER stress regulation.^34^

Interestingly, we also observed altered gene expression relating to amino acid metabolism and transport in SIRT^intKO^ mice at both 6 and 24 hours after PHx, which has been described as key for supporting the transition from quiescence to cell cycle activation and proliferation in hepatocytes.^35^^,^^36^

The liver undergoes metabolic reprogramming during regeneration,^36^^,^^37^ which is associated with heavy metabolic demands and changes. Particularly, lipid accumulation and further oxidation release energy to support hepatocyte proliferation during liver regeneration.^36^ GO and DEGs analyses at 24 hours after PHx highlighted profound differences in the regulation of lipid metabolism, including increased expression of key lipid metabolism regulators in WT livers compared with SIRT^intKO^ livers, including *Srebf1* (lipogenesis), *Star* (cholesterol trafficking), and *Fabp5* (fatty acid binding). The increased expression in WT mice of *Slc27a5*, key for fatty acid transport critical for liver regeneration in mice after PHx,^38^ along with the reduced expression of *Dgat1*, a key regulator of triglyceride synthesis with demonstrated effects on influencing tumor cell proliferation,^39^ in SIRT^intKO^ mice at 24 hours after PHx support the altered lipid metabolism in intestinal SIRT1-deficient mice that may contribute to the reduced proliferation we observed.

After the priming and proliferative phase, liver regrowth is tightly regulated by cell-cycle termination signals that prevent uncontrolled hepatocyte proliferation and excessive liver growth during liver regeneration. Senescence is a cellular mechanism that mediates cell-cycle arrest, playing a key part in the termination of hepatocyte proliferation through the upregulation of p21,^20^ which has been described to occur after the peak of hepatocyte proliferation at 48 hours after PHx.^40^^,^^41^ Although this response is aimed at arresting the cell cycle and avoiding abnormal proliferation, excessive senescence impairs liver regeneration, as described by Ferreira-Gonzalez and colleagues.^42^ In light of these observations, our results support that early (24 hour) and sustained (48 hour) p21 expression in hepatocytes in SIRT^intKO^ mice contributes to reduced hepatocyte proliferation after PHx.

Previous studies described the association between senescence and the activation of stem cells in the liver during the response to injury.^20^ When hepatocyte proliferation is compromised, the liver has the capacity to regenerate from liver progenitor cells (LPCs), also called oval cells due to their shape, that proliferate and differentiate into hepatocytes.^23^^,^^43^ Our results show that early senescence associates with expansion of LPCs in the liver parenchyma of SIRT^intKO^ mice at 24 and 48 hours after PHx, which contribute to the regeneration of the liver overall reaching a comparable mass compared with WT mice at 10 days after surgery. Interestingly, a previous study showed that MDR1 is highly expressed in regenerative areas of the liver where progenitor cells are also highly present.^29^ In line with this, we also observed that SIRT^intKO^ mice show high MDR1 gene expression and increased presence of LPCs in the liver after PHx.

Ultimately, our results showing that the intestinal-specific activation of FXR with Fex restores hepatocyte proliferation in SIRT^intKO^ mice at comparable levels to WT mice support our hypothesis that SIRT1 is an upstream regulator of FXR function during liver regeneration. We and others have previously described the beneficial effects of Fex in modulating the liver response to injury.^27^^,^^28^ Our results support the view that the benefits of Fex-modulation of FXR expands to liver regeneration after PHx.

Together, our results establish that SIRT1 is the upstream regulator of intestinal FXR/FGF15 signaling and is therefore a key regulator of hepatocyte proliferation and liver regeneration after PHx. Our results contribute to improving our mechanistic understanding of the contribution of intestinal signals in controlling liver regeneration and point to intestinal SIRT1 as potential therapeutic target to improve the liver regenerative response to injury.

## Material and Methods

### Experimental Procedures in Animals

All experimental procedures were approved by the Animal Welfare and Ethical Review Body (University of East Anglia) and performed at the Disease Modelling Unit (University of East Anglia). All procedures were carried out following the guidelines of the National Academy of Sciences (National Institutes of Health, publication 86-23, revised 1985) and were performed within the provisions of the Animals (Scientific Procedures) Act 1986.

For all experiments shown here, we used 8- to 12-week-old male mice of C57/Bl6 background. Intestinal-specific *Sirt1* knockout mice (herein SIRT^intKO^) were generated by crossing mice carrying a Villin Cre-recombinase with mice containing floxed sites flanking exon 4 of *Sirt* gene (B6;129-Sirt1tm1Ygu/J; Jackson Laboratories). Mice carrying loxP-flanked *Sirt1* alleles were used as controls when compared with *Villin-Cre*-containing *Sirt1* floxed mice (WT). Mice underwent PHx of 70% of the liver by resection of the left lateral and median lobes^44^ following the LASA Guiding Principles for Preparing for Undertaking Aseptic Surgery (2010) under United Kingdom Home Office approval (PP9417531 to Naiara Beraza).

### Determination of Liver Damage in Serum

ALT and AST were determined in serum samples using the Randox Daytona+ analyzer (RX Daytona+) following the manufacturer’s instructions, as we previously described.^11^

### Histology, Immunohistochemistry, and Immunofluorescence

Liver tissues were fixed in 10% neutral buffered formalin, embedded in paraffin, and further sectioned for pathologic analysis and IHC as we previously described.^11^^,^^28^^,^^45^ For pathologic analysis, liver sections were dewaxed, hydrated, and stained using hematoxylin and eosin to determine parenchymal status and visualize areas of necrosis. Slides were imaged using brightfield on a BX53 upright microscope (Olympus) with an Olympus DP74 color camera and a pT100 LED transmitted light source (CoolLED). Five to 10 fields of view per sample were analyzed using open-source FIJI software,^46^ as we described previously,^11^^,^^45^ and necrotic areas were represented as the percentage relative to total area per field.

IHC was performed in paraffin-embedded liver sections using an anti-BrdU antibody (AB2284-Abcam), p21 (Abcam), SOX9 (Cell Signaling), and CK19 (TROMAIII, Developmental Studies Hybridoma Bank) diluted in antibody diluent (Dako; S0809), after which sections were incubated with EnVision^+^ polymer horseradish peroxidase (HRP)-labeled anti-mouse (Dako; K4001), anti-rabbit (Dako; K4003), an anti-rat HRP- or anti-sheep HRP-labeled secondary antibody. IHC was developed using the DAB^+^ chromogen system (Dako; K3468), after which the nuclei were counterstained with hematoxylin. Slides were imaged on a BX53 and analyzed using FIJI as described above.

Sodium citrate antigen retrieval was used for immunofluorescence in paraffin liver sections using an anti–p-Histone H3 (Cell Signaling Technologies) and anti-rabbit Cy3-labeled secondary antibody (Jackson ImmunoResearch). Slides were mounted with a 4′,6-diamidino-2-phenylindole mounting solution (Vector Laboratories; H-1200) to stain cell nuclei. Fluorescent microscopic imaging was performed using an AxioImager M2 (Zeiss) with the AxioCam mRM monochrome camera and standard light source and filter sets supplied. Images were analyzed using the ZenBlue Software (Zeiss) and or FIJI.^46^

### RNA Isolation and RNA Sequencing Analysis

RNA was isolated from liver samples using QiAzol lysis Reagent (Qiagen; 79,306). Library preparation and sequencing were done by Macrogen Ltd. Briefly, stranded messenger RNA libraries were prepared using the TruSeq Stranded mRNA Library Prep Kit (Illumina), following the manufacturer’s protocol. Sequencing was performed on the Illumina NovaSeq X platform, generating 100 base pair paired-end reads. Each library was sequenced to a depth of approximately 40 million paired-end reads.

Raw sequencing reads were processed using the nf-core/rnaseq pipeline with default settings. This pipeline includes adapter trimming, alignment using STAR, and transcript quantification using Salmon.

Gene-level count matrices were imported into R and analyzed using the differential gene expression analysis tool (DESeq2 package; v1.38.3). Genes with low counts were filtered out, and a DESeqDataSet object was constructed using the design formula:

∼genotype+timepoint+genotype:timepoint.

Normalization and dispersion estimation were performed using DESeq2’s standard workflow. Differential expression was assessed with Wald tests, and log2 fold changes were shrunk using the lfcShrink() function. Genes with adjusted *P* < .05 and absolute log2 fold change > 1 were considered differentially expressed.

Functional enrichment analysis of DEGs was performed using GO with the cluster Profiler package. DEGs were categorized as shared between WT and KO, unique to WT, or unique to KO.

Enriched GO terms were visualized using custom ggplot2 dot plots, and top terms were manually curated for biological relevance. Differential expression analyses were performed separately for each genotype (0 vs 6 hours, and 0 vs 24 hours) to define early and late transcriptional responses.

### Correlation Analyses

Correlation analyses were performed to assess associations between BA levels, gene expression, and liver injury markers. Serum liver injury markers (AST and ALT) were correlated with total BA concentrations using Spearman correlation. For transcriptomic analyses, normalized gene expression values obtained from RNA-seq (rlog-transformed counts) were correlated with BA levels using the same method. When correlations involved multiple BA species, *P* values were adjusted to account for multiple testing.

### Western Blot Analysis

Proteins were extracted from snap frozen liver tissues using radioimmunoprecipitation assay buffer containing 50 mM Tris-HCL, 150 mM NaCl, 0.1% sodium dodecyl sulfate, 2 mM EDTA, 5% sodium deoxycholate, 1% Igepal 630, 1 mM phenylmethylsulfonyl fluoride, and protease inhibitor (Sigma-Aldrich; 4,693,124,001). Proteins were resolved in sodium dodecyl sulfate–polyacrylamide gels and transferred to nitrocellulose membranes (Whatman; WHA1541185). Membranes were probed with SIRT1 (Cell Signaling), cyclin D1 antibody (Santa Cruz), p-STAT3 (Cell Signaling Technologies), FXR (Santa Cruz), and FGF15 (Santa Cruz). β-Actin and glyceraldehyde-3-phosphate dehydrogenase were used as loading controls. Anti-rabbit IgG-HRP–linked (Cell Signaling Technologies; 7074S) or anti-mouse IgG-HRP–linked (Cell Signaling Technologies; 7076S) were used as secondary antibodies. Images of the blots were taken using ChemiDoc MP imaging system (Bio-Rad). Quantification was performed by normalizing the target protein signal intensity with housekeeping proteins (glyceraldehyde-3-phosphate dehydrogenase) using ImageLab software (BioRad).

### Bile Acid Extraction and Analysis

A portion of sample (approximately 50 mg) was taken into a screw cap tube along with ceramic beads; 1 mL of 90% v/v methanol and 25 μL of 40 μg/mL d4-GCA were added and homogenized for 30 seconds at 6000 rpm. The slurry was centrifuged at maximum speed for 10 minutes at 4 °C. The supernatant was passed through a Waters Oasis PRiME HLB 30 milligram μElution 96-well plate (P/M 186,008,054). The cleaned-up extracts in the capture plate were analyzed using high-performance liquid chromatography–mass spectrometry operated in multiple reaction monitoring mode. Each sample (5 μL) was analyzed using a Waters Acquity ultra-performance liquid chromatography coupled to a Xevo TQ Absolute triple quadrupole mass spectrometer. High-performance liquid chromatography was achieved using a binary gradient of solvent A (water + 5 mM ammonium acetate + 0.012% formic acid) and solvent B (methanol + 5 mM ammonium acetate + 0.012% formic acid) at a constant flow rate of 900 μL/min. Separation was made using a Supelco Ascentis Express C18 150 × 4.6, 2.7-μm column maintained at 40 °C. Injection was made at 50% B and held for 2 minutes, ramped to 95% B at 20 minutes, and held until 24 minutes. The column was equilibrated to initial conditions for 5 minutes. The mass spectrometer was operated in electrospray negative selected ion mode. Quantification was applied using Waters TargetLynx software to integrate detected peak areas relative to the deuterated internal standards.

### Statistical Analyses

Statistical analyses were performed using GraphPad Prism software Version 10.4.1. Statistical differences between groups were determined by unpaired, 2-tailed Student’s *t* test with Welch’s correction when comparing 2 groups at 1 single timepoint. When comparing 2 groups at multiple timepoints, differences were determined using 2-way analysis of variance with the Holm–Sidak test. RNA-seq data were preprocessed using the Nextflow pipeline, and differential expression analysis was performed with DESeq2, considering genes with adjusted *P* value < .05 and |log_2_ fold change| >1 as significant. Data are shown as mean ± standard error of the mean. Significance is indicated by: ∗*P* < .05, ∗∗*P* < .01, ∗∗∗*P* < .001, ∗∗∗∗*P* < .0001.
